# Supplementary material for: The genome-wide multi-layered architecture of chromosome pairing in early Drosophila embryos
Source: Nat Commun. 2019 Oct 3;10:4486. doi: 10.1038/s41467-019-12211-8 (PMC6776651; doi:10.1038/s41467-019-12211-8)
Supplement: Supplementary file 1 — Supplementary Information [file 41467_2019_12211_MOESM1_ESM.pdf]

Supplementary Information

The genome-wide multi-layered architecture of chromosome pairing in early *Drosophila* embryos

Erceg *et al*.

SUPPLEMENTARY NOTE 1

The choice of two parental *Drosophila* lines

To ensure high quality and accuracy of variant calling, we used only those *Drosophila melanogaster* Genetic Reference Panel (DGRP) lines[1] whose sequence coverage with Illumina technology was at least 20x. Variant annotation for each fly line with at least 20x sequence coverage was extracted from Freeze 2 Release (https://www.hgsc.bcm.edu/arthropods/drosophila-genetic-reference-panel; freeze2.bins.vcf.gz), and a count of the number of SNVs against reference genome (VCFTtools, vcf –stats function)[2] was annotated. We proceeded with the top 5 and bottom 5 ranked *Drosophila* lines by filtering their variant annotations to keep only homozygous SNVs, and then performed pairwise comparison (vcftools --vcf DGRP-x_homSNV.vcf --diff DGRP-y_homSNVs.vcf). Upon inspection of the output file from the pairwise comparison (out.diff.site_in_files), which contained information on both common and unique homozygous SNVs between two lines, we selected two parental lines, DGRP-057 and DGRP-439, that differed by most homozygous, fixed SNVs.

Calculation of insulation scores

The tracks of contact insulation score were calculated using the package cooltools insulation (https://github.com/mirnylab/cooltools). The method used in the package was based on the algorithm described in[3], and modified in[4].

SUPPLEMENTARY FIGURES

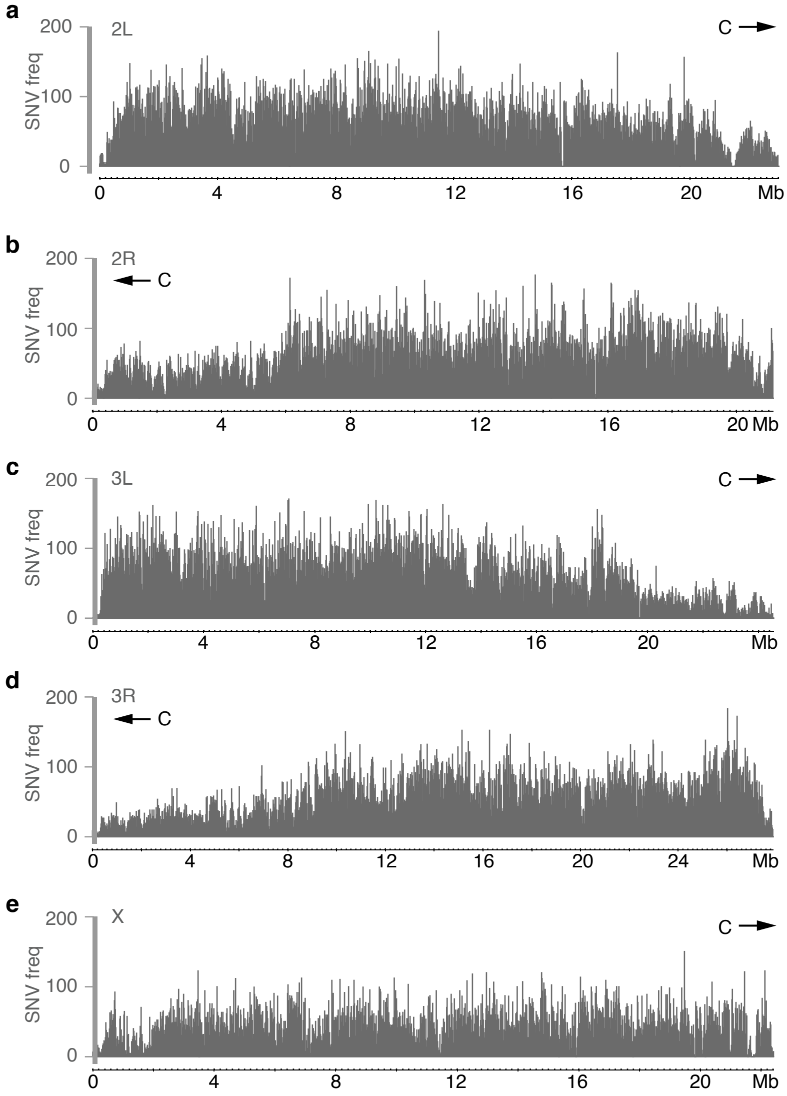

Supplementary Fig. 1. Distribution of SNVs in the F1 hybrid *Drosophila*.

a-e, The distribution of SNVs along 2L (**a**), 2R (**b**), 3L (**c**), 3R (**d**), and X (**e**) at 4 kb resolution. Note that the SNV frequency decreases in pericentromeric regions. Arrow points towards direction where centromere (C) should be located.

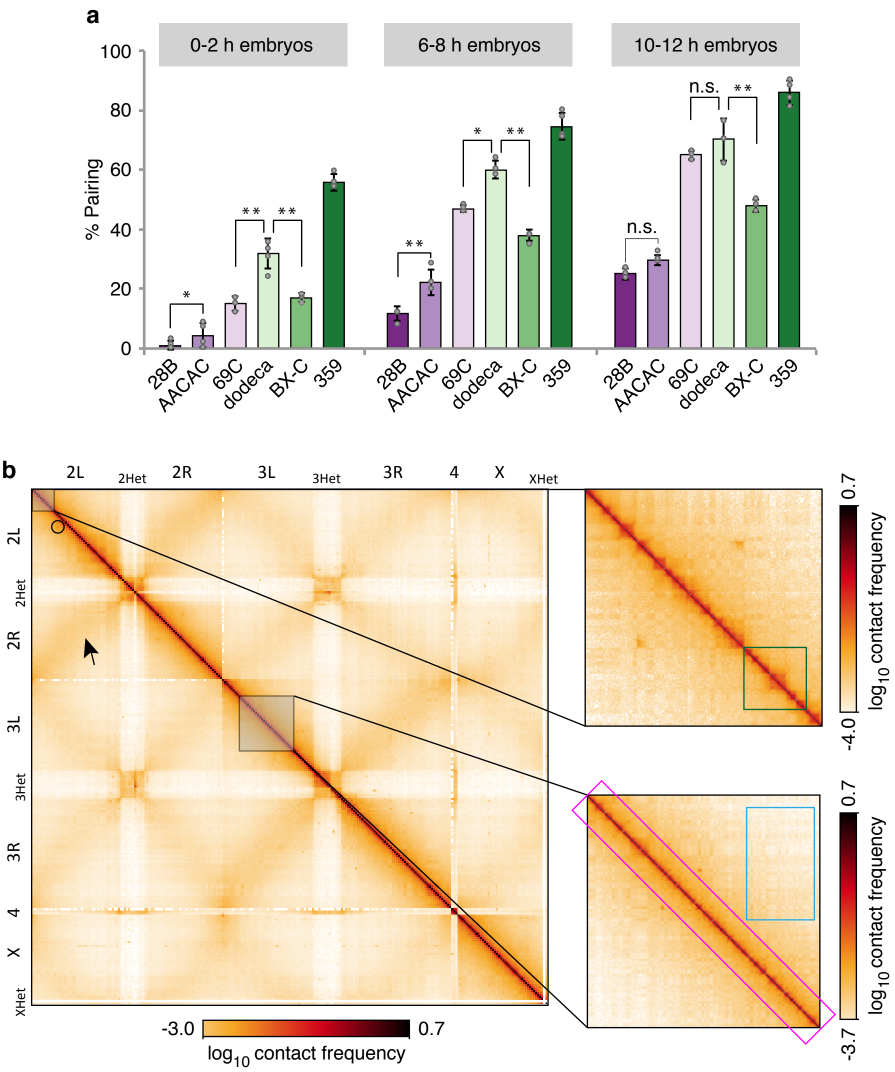

Supplementary Fig. 2. Assessment of homolog pairing by FISH, and of Hi-C data quality in the F1 hybrid embryos.

a, Levels of homolog pairing increase steadily as development progresses in the F1 hybrid embryos. Percentage of nuclei showing paired loci at the FISH targets from Fig. 2d in 0-2 h, 6-8 h, and 10-12 h embryos (error bars, standard deviation of at least 3 replicates; n ≥100 nuclei/replicate). Nuclei are considered paired nuclei when FISH signals are ≤0.8 μm (center-to-center distance) apart. Pairing levels at heterochromatic targets are significantly higher than at euchromatic that lie on the same chromosome during 0-2 h, 2-4 h (Fig. 2e), and 6-8 h. However, later during development, at 10-12 h, levels become more similar (*P<0.05, **P<0.0001, n.s. not

significant; Fisher's two-tailed exact). Source data are provided as a Source Data file.

b, Genome-wide Hi-C map of the 2-4 h F1 hybrid embryos mapped to the reference dm3 genome. Greyed out boxes, zoomed-in views on 2L and 3L. Features such as central *cis* diagonal (pink box), domains (green box), plaid-patterned compartments (blue box), interaction peaks (circle), and contacts consistent with Rabl (arrow) are indicated. The maps were visualized using the HiGlass browser[5].

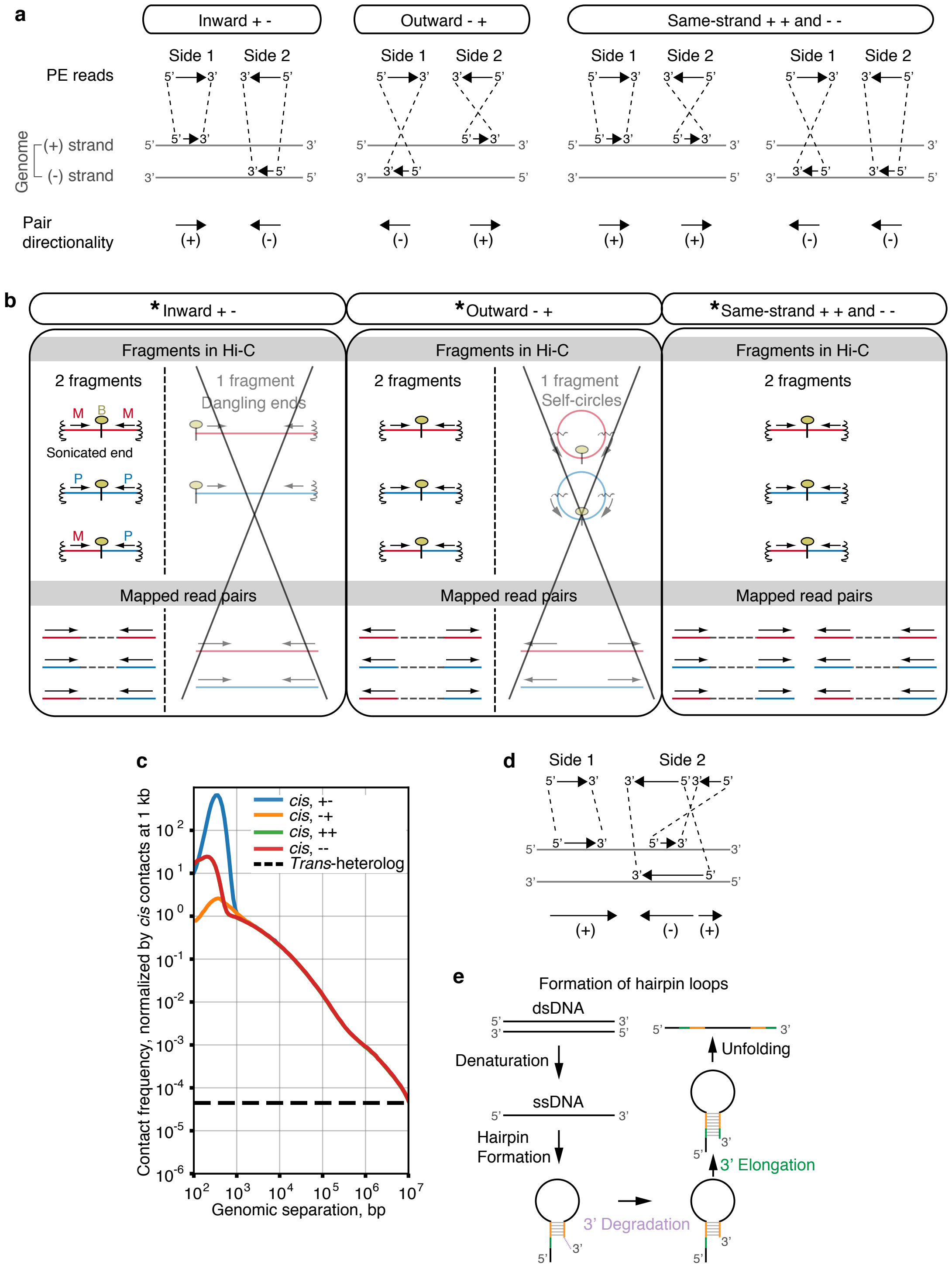

Supplementary Fig. 3. The classification of Hi-C byproducts.

a, Mapped read pairs can be classified based on relative locations and directions of both sides of a read pair into three types: inward, outward, and same-strand. **b,** As adapted from[6], Hi-C byproducts manifest themselves as Hi-C pairs of a specific

directionality. These byproducts appear as inward or outward pairs, and should not appear in *trans* (crossed out) (asterisk, see Methods for other sources of contamination). Red, maternal (M); blue, paternal (P); yellow ellipse, biotin; wiggly vertical line, sonicated ends. **c,** Contact frequency plotted against genome separation using ≤ 1 SNV per read, and split by read pair directionality into inward, outward, and same-strand. **d,** A common mapping pattern of read pairs observed in our data, with one read fully aligned to one genomic locus, and the other side split into two alignments of opposite directionality. **e,** Formation of hairpin loops, as suggested to be the source of contamination among same-strand reads[7,8]. Upon denaturation, palindromic sequence of ssDNA (orange) may form hairpin structure. Unannealed 3' ends (purple) may be degraded, and refilled with sequence based on 5'end (green) during generation of the Hi-C library.

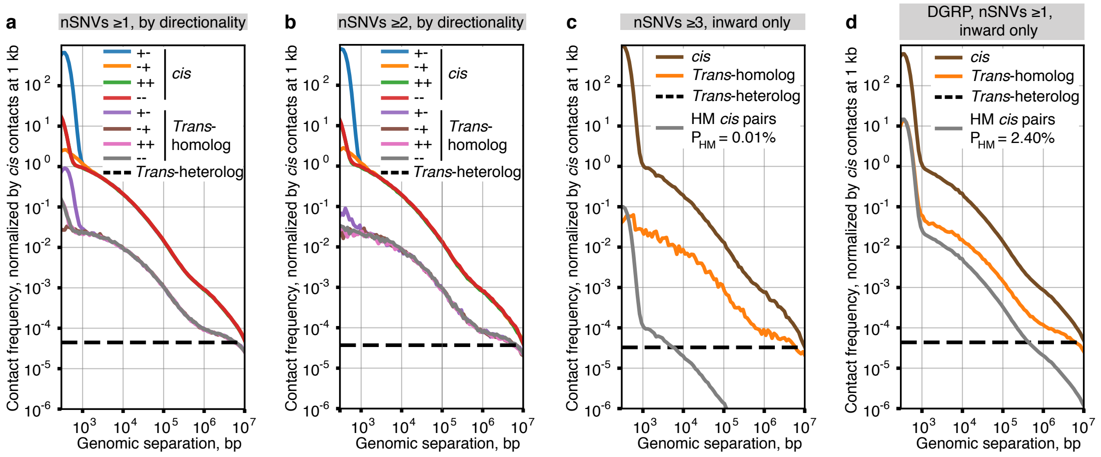

Supplementary Fig. 4. Homolog misassignment depends on the number and quality of SNVs.

a-b, Contact frequency plotted against genome separation, and split by read pair directionality to inward, outward, and same-strand for pairs containing at least (**a**) 1 SNV or (**b**) 2 SNVs per read. Requiring ≤2 SNVs per read removed a characteristic enrichment of inward *trans*-homolog contact frequency at s<1 kb, which suggested that this enrichment in unfiltered data was caused by homolog misassignment (HM) of *cis* pairs. **c,** Contact frequency for inward read pairs with at least 3 SNVs, with no sequence mismatches allowed (P_{HM}=0.01%). **d,** Contact frequency for inward read pairs mapped using the DGRP SNV annotations[1] requiring at least 1 SNV per read (P_{HM}=2.4%). **a-d,** Contact frequencies for chromosomes 2 and 3 normalized by *cis* contact frequency at 1 kb. Dashed black line, average *trans*-heterolog contact frequency; P_{Hm}, probability of homolog misassignment.

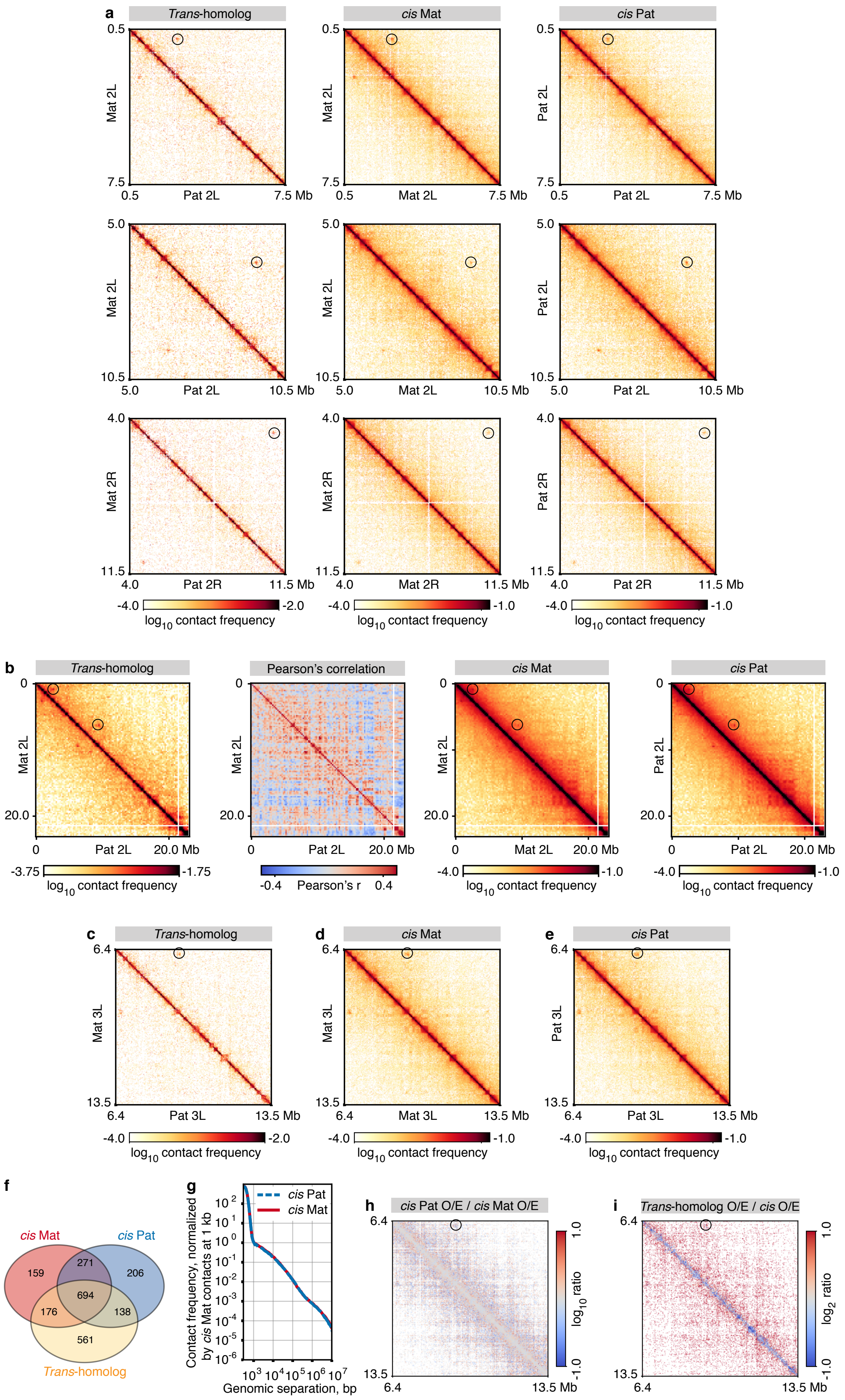
a
Trans-homolog
cis Mat
cis Pat
Mat 2L
Pat 2L
Mat 2R
Pat 2R
log10 contact frequency
b
Pearson's correlation
Pearson's r
c
d
e
Mat 3L
Pat 3L
f
cis Mat
cis Pat
Trans-homolog
159
271
206
694
176
138
561
g
Contact frequency, normalized by cis Mat contacts at 1 kb
Genomic separation, bp
h
cis Pat O/E / cis Mat O/E
log10 ratio
i
Trans-homolog O/E / cis O/E
log2 ratio

Supplementary Fig. 5. Haplotype-resolved Hi-C maps suggest highly structured *trans*-homolog features resemble analogous *cis* features.

a, *Trans*-homolog, *cis* maternal, and *cis* paternal maps showing interaction peaks on 2L and 2R. **b,** *Trans*-homolog map with the Pearson's correlation for the plaid-patterned *trans*-homolog compartments, including matching *cis* maternal and *cis* paternal maps on 2L. **c,** *Trans*-homolog, **d,** *cis* maternal, and **e,** *cis* paternal maps of matching ~7 Mb regions on 3L. **f,** Venn diagram shows the extent of overlap among *trans*-homolog boundaries, maternal and paternal *cis*-boundaries. **g,** Contact frequency plotted against genome separation for chromosomes 2 and 3 normalized by the *cis* maternal contact frequency at 1 kb. Note that normalizing by the *cis* paternal contacts at 1 kb gave essentially the same result. **h,** The ratio of *cis* Pat/*cis* Mat Hi-C maps from d and e indicates the *cis* contact patterns of two homologs are highly concordant. **i,** The ratio of *trans*-homolog/average *cis* maps from c-e suggests that pairing resembles *cis* contacts, albeit with lower interactions in some regions (dark blue). **a-e, h-i,** Interaction peaks are encircled. The maps displayed (3L:6.4-13.5Mb) represent a zoomed-out from the maps (3L:9.85-11.6Mb) in Fig. 3i-m.

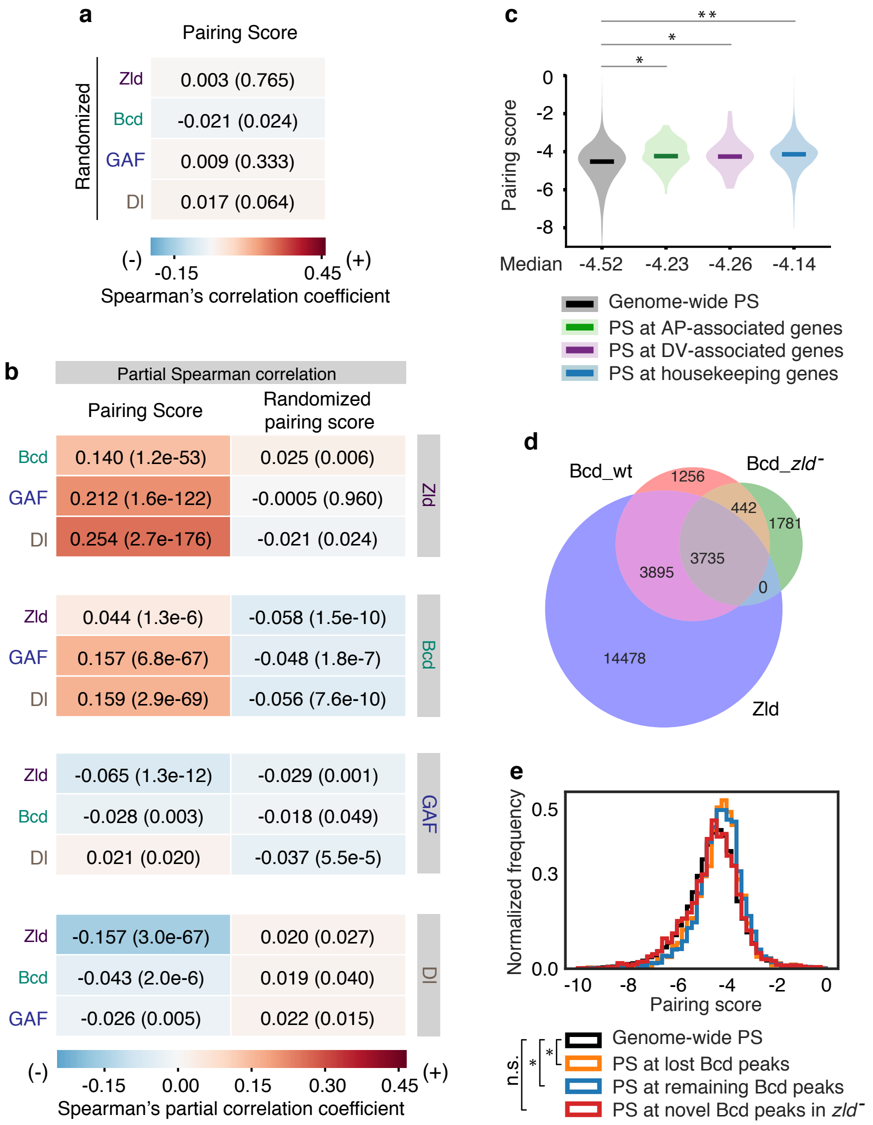

Supplementary Fig. 6. Zld-mediated opening of chromatin affects Bcd's relation to pairing.

a, Correlation analyses between the PS values and randomized 200 kb chunks of the binding profiles of Zld, Bcd, GAF, and Dl as determined using pairwise Spearman correlation. Spearman correlation coefficients indicated in each box and by a heatmap; P-values in parentheses. **b,** Partial Spearman correlation analyses showing, for instance, whether the correlation between a feature in a column (the PS or random PS values) and the binding of Zld (grey box on the right side) is affected after removal of any contribution of features displayed in the rows, such as the binding of

Bcd, GAF, and Dl. Similar to assessments pertaining to Zld, partial correlation analyses were performed for each of the other factors highlighted in grey boxes. Partial Spearman correlation coefficients indicated in each box and by a heatmap; P-values in parentheses. **c,** Distribution of PS values at genes associated with AP or DV patterning[9,10], as well as at housekeeping genes[11,12] compared to the genome-wide PS values (*$P<1.90 \times 10^{-2}$, **$P=8.18 \times 10^{-84}$; Mood's median test; medians indicated with horizontal lines). **d,** Venn diagram shows the extent of overlap among peaks of Zld[13], wild-type Bcd (Bcd_wt)[14], and Bcd in Zld-depleted embryos (Bcd_*zld*$^{-}$)[14]. **e,** Distribution of Bcd peaks in Zld-depleted embryos relative to the PS values. Genome-wide PS represents the average pairing score over the genome. (*$P<1.21 \times 10^{-68}$, n.s. not significant; Mood's median test).

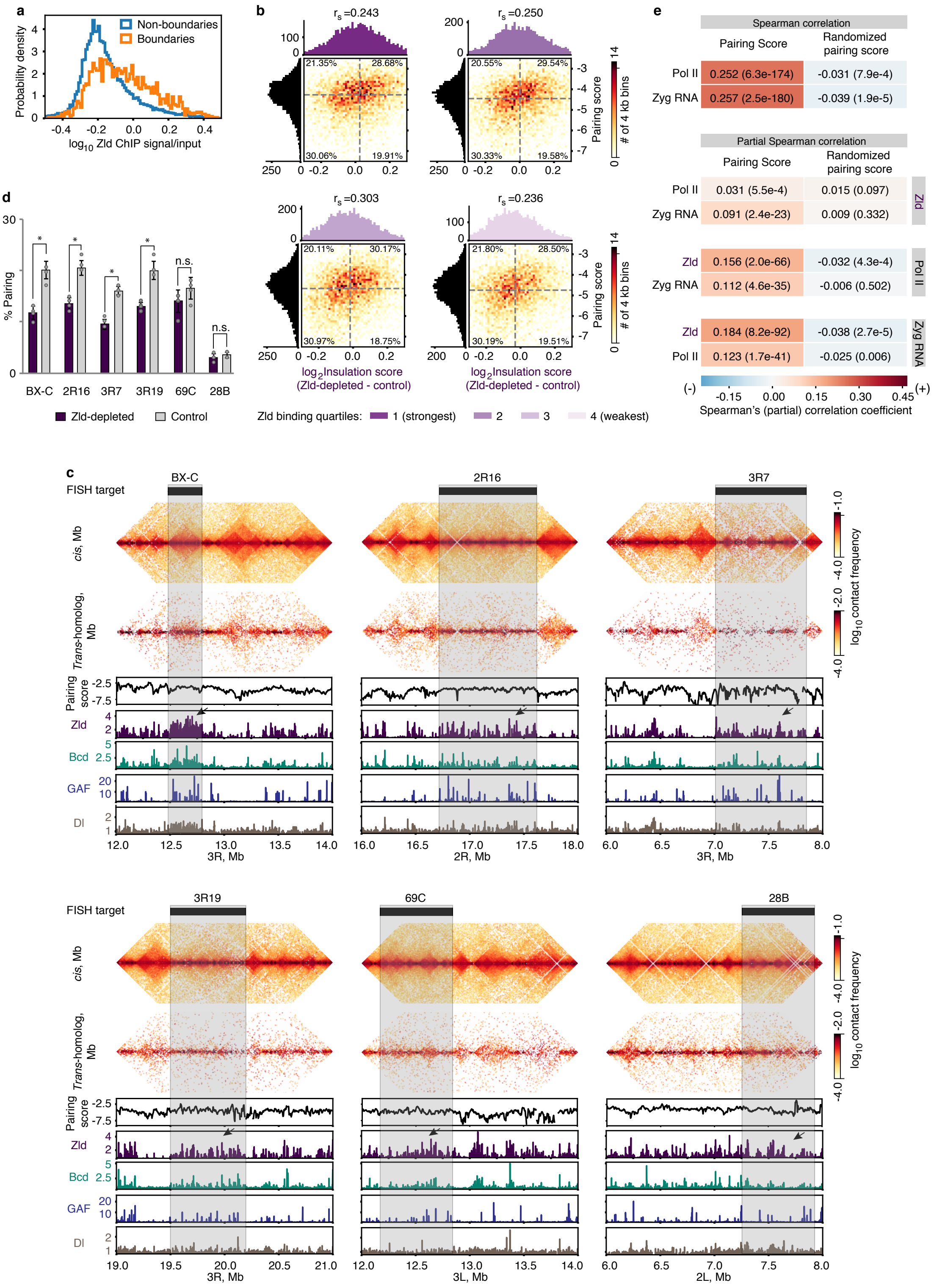

Supplementary Fig. 7. Zld-dependent genome activation is associated with homolog pairing.

a, Distribution of Zld signal suggests that domain boundaries are more likely to have strong Zld binding than non-boundaries ($P<10^{-10}$, Mood's median test). **b,** Distribution of the difference in insulation scores between Zld-depleted and control nc14 embryos relative to the pairing score, stratified by the Zld binding quartiles (also depicted as fitted ellipses in Fig. 4d). Dashed lines, medians; r_s, Spearman's correlation coefficient. **c,** *Cis* (upper panels) and *trans*-homolog (second panels from the top) contact maps. Lower panels, pairing score (PS) calculated using a 28 kb window at 4 kb resolution (black), the ChIP-seq profiles of Zld (dark purple)[13], Bcd (green)[14], GAF (blue)[15], and Dl (brown)[13]. Grey boxes, locations of FISH targets; arrows, Zld signal at FISH targets. **d,** Percentage of nuclei showing paired loci for Zld-depleted and control embryos (cycle 14; error bars, standard deviation of at least 4 replicates; n ≥100 nuclei/replicate; *, $P<2.56 \times 10^{-3}$, n.s. not significant, Fisher's two-tailed exact). Source data are provided as a Source Data file. **e,** Correlation analyses. Spearman correlation: Analyses between the PS values and two features, the RNA Pol II binding[13] and nascent zygotic gene expression[16], as determined using pairwise Spearman correlation. Control, randomized 200 kb chunks of the PS. Partial Spearman correlation: Given the role of Zld in chromatin opening and genome activation, the observed pairwise correlations with the PS values are influenced by co-correlation when controlling for other features, such as Zld, RNA Pol II, and nascent zygotic (zyg) gene expression. (Partial) Spearman correlation coefficients indicated in each box and by a heatmap; P-values in parentheses.

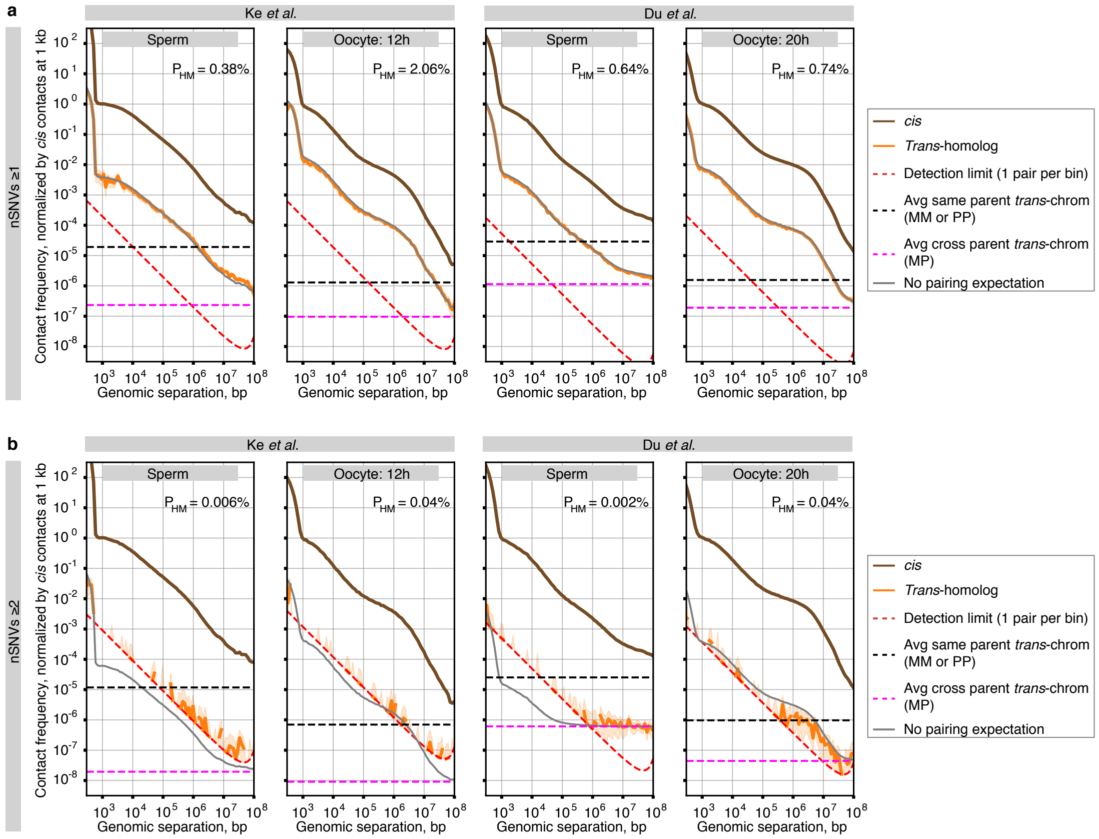

Supplementary Fig. 8. A stringent filtering of haplotype-resolved Hi-C datasets from mouse gametes removes *trans*-homolog pairs.

a-b, Contact frequency plotted against genome separation in Ke *et al*.[17] and Du *et al*.[18] gamete Hi-C datasets, requiring at least (**a**) 1 or (**b**) 2 SNVs per read. Shaded area, 95% confidence intervals calculated via Poisson resampling. Dotted lines, average *trans* contact frequency as a function of distance between the same parent of origin chromosomes (black; MM or PP), or between maternal and paternal chromosomes (pink; MP). Grey line, expected *trans*-homolog contact frequency as a function of distance without pairing, but in presence of homolog misassignment (HM). No sequence mismatches allowed for all read pairs. Maternal (M), and paternal (P) chromosomes. P_{Hm}, probability of homolog misassignment.

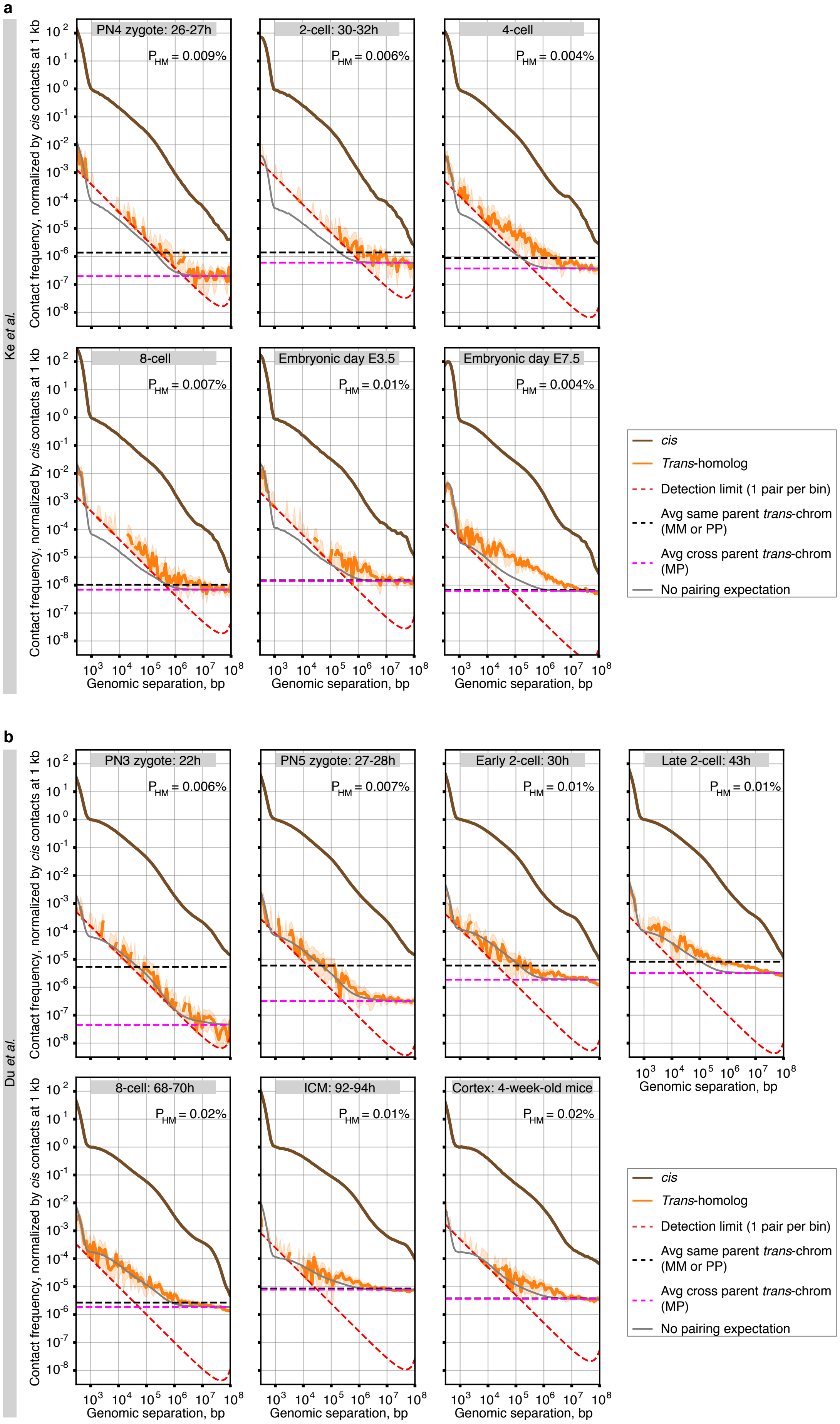
a
Ke et al.
Contact frequency, normalized by cis contacts at 1 kb
PN4 zygote: 26-27h
P_{HM} = 0.009%
2-cell: 30-32h
P_{HM} = 0.006%
4-cell
P_{HM} = 0.004%
8-cell
P_{HM} = 0.007%
Embryonic day E3.5
P_{HM} = 0.01%
Embryonic day E7.5
P_{HM} = 0.004%
Genomic separation, bp
cis
Trans-homolog
Detection limit (1 pair per bin)
Avg same parent trans-chrom (MM or PP)
Avg cross parent trans-chrom (MP)
No pairing expectation
b
Du et al.
PN3 zygote: 22h
P_{HM} = 0.006%
PN5 zygote: 27-28h
P_{HM} = 0.007%
Early 2-cell: 30h
P_{HM} = 0.01%
Late 2-cell: 43h
P_{HM} = 0.01%
8-cell: 68-70h
P_{HM} = 0.02%
ICM: 92-94h
P_{HM} = 0.01%
Cortex: 4-week-old mice
P_{HM} = 0.02%

Supplementary Fig. 9. Haplotype-resolved Hi-C datasets representing hybrid mouse embryos show a minor population of *trans*-homolog read pairs.

a-b, Contact frequency plotted against genome separation in (**a**) Ke *et al.*[17] or (**b**) Du *et al.*[18] datasets, requiring at least 2 SNVs per read. Shaded area, 95% confidence intervals calculated via Poisson resampling. Dotted lines, average *trans* contact frequency as a function of distance between the same parent of origin chromosomes (black; MM or PP), or between maternal and paternal chromosomes (pink; MP). Grey line, expected *trans*-homolog contact frequency as a function of distance without pairing, but in presence of homolog misassignment (HM; no pairing expectation). No mismatches allowed for all read pairs. Maternal (M), and paternal (P) chromosomes. P_{Hm}, probability of homolog misassignment.

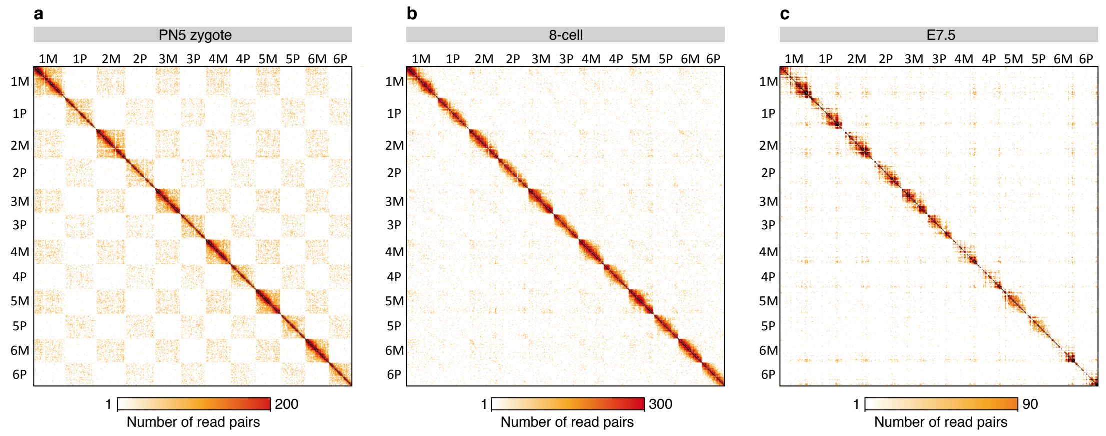

Supplementary Fig. 10. Segregation of parental genomes reduces during developmental progression.

a-c, Haplotype-resolved Hi-C maps for the first 6 chromosomes with 2 SNVs per read are displayed using the HiGlass browser[5] for (**a**) PN5 zygote and (**b**) 8-cell embryos from Du *et al*.[18], and (**c**) E7.5 embryos from Ke *et al*.[17]. Maternal (M); paternal (P); M, C57BL/6N; P, PWK/PhJ for Du *et al*. datasets, while M, C57BL/6J; P, DBA/2J mice strain for Ke *et al*. dataset.

SUPPLEMENTARY TABLES

Chrom Name	Chrom Size	Indels			SNVs			
		Hom	HetMat	HetPat	Hom	HetMat	HetPat	Het per kb
**2L**	23,011,544	9,415	5,820	5,910	68,091	66,894	70,989	5.992
**2R**	21,146,708	8,828	5,144	5,396	60,606	55,659	58,689	5.407
**3L**	24,543,557	10,374	6,068	5,599	73,011	67,299	64,261	5.360
**3R**	27,905,053	9,676	6,494	6,402	65,627	66,777	68,709	4.855
**4**	1,351,857	136	1	4	929	2	15	0.013
**X**	22,422,827	5,193	8,405	8,386	38,063	40,674	44,055	3.779

Supplementary Table 1. Variant annotations for maternal (DGRP-057) and paternal (DGRP-439) lines.

Numbers of indels and SNVs are shown for each chromosome in both homozygous (Hom) and heterozygous (Het) scenario. Maternal (Mat); paternal (Pat).

	Replicate 1	Replicate 2
**Total**	256,988,962	256,274,483
**Total_unmapped**	53,747,071	50,506,661
**Total_ss_mapped**	37,369,722	37,037,449
**Total_mapped**	165,872,169	168,730,373
**Duplicates**	23,641,775	21,411,225
**Non-duplicates**	142,230,394	147,319,148
cis	131,681,245	134,880,524
trans	10,549,149	12,438,624
***cis/trans* ratio**	12.5	10.8
**Mappability (%)**	55.3	57.5

Supplementary Table 2. Statistics summary for the dm3 reference mapping and duplicate removal.

The number of read pairs is provided for each biological replicate. Total raw read pairs ('Total') include unmapped read pairs with multiple or low score alignments ('Total_unmapped'), and read pairs where only single side ('Total_ss_mapped') or both sides are mapped ('Total_mapped')[6]. Total mapped read pairs contain non-duplicates and discarded PCR duplicates. Non-duplicates are subdivided into *cis* and *trans* read pairs. Portion of non-duplicates with respect to total raw read pairs is indicated as mappability. The average mappability between two replicates is ~56%.

	SNVs from this study		SNVs from Mackay *et al.* 2012	
	**Replicate 1**	**Replicate 2**	**Replicate 1**	**Replicate 2**
**Total**	256,988,962	256,274,483	256,988,962	256,274,483
**Total_unmapped**	183,409,873	180,413,502	174,706,472	171,547,794
**Total_ss_mapped**	45,654,919	47,956,696	57,145,342	59,638,376
**Total_mapped**	27,924,171	27,904,286	25,137,148	25,088,313
**Duplicates**	3,533,675	3,245,230	2,220,900	1,959,943
**Non-duplicates**	24,390,496	24,659,056	22,916,248	23,128,370
cis	23,076,479	23,130,134	21,265,473	21,243,109
trans	*1,314,017	*1,528,922	1,650,775	1,885,261
***cis/trans* ratio**	17.6	15.1	12.9	11.3
**Mappability (%)**	9.5	9.6	8.9	9.0

Supplementary Table 3. Statistics summary for the haplotype-resolved mapping and duplicate removal.

Haplotype-resolved mapping is performed using SNVs defined in this study or in Mackay *et al*. 2012[1]. The number of read pairs is provided for each biological replicate. Total raw read pairs ('Total') include unmapped read pairs with multiple or low score alignments comprising also reads without SNVs ('Total_unmapped'), and read pairs where only single side ('Total_ss_mapped') or both sides are mapped ('Total_mapped'). Total mapped read pairs contain non-duplicates and discarded PCR duplicates. Non-duplicates are subdivided into *cis* and *trans* read pairs. Portion of non-duplicates with respect to total raw read pairs is indicated as mappability. *Given that the number of *trans*-homolog read pairs is 497,766 (Replicate 1) and 528,169 (Replicate 2), 36.1% of all *trans* read pairs are *trans*-homolog.

Probe set	Chr	Start (dm3)	Stop (dm3)	Target size/kb	# Oligos	Probes/kb
89D-89E/ BX-C	3R	12,482,502	12,797,965	315	2,394	7.6
2R16	2R	16,714,862	17,618.516	904	14,833	16.4
3R7	3R	7,011,837	7,856,096	844	13,632	16.1
3R19	3R	19,495,483	20,195,233	700	11,458	16.4
69C	3L	12,170,682	12,844,681	674	10,000	14.8
28B	2L	7,256,488	7,936,487	680	10,000	14.7

Supplementary Table 4. Information on Oligopaint probe sets.

For each Oligopaint probe set information such as the span in the genome with start and stop coordinates, target size, number of oligos, and probe density are given.

Probe set	Oligos	Sequence 5'->3'
89D-89E/ BX-C	Fw	CACACGCTCTCCGTCTTGGCCGTGGTCGATCAGTATCGTGCAAGGGTGAATGC
	Rev	TAATACGACTCACTATAGGGGAGCAGTCACAGTCCAGAAGG
	Sec oligo	/5Alex647N/TGATCGACCACGGCCAAGACGGAGAGCGTGTG/3AlexF647N/
2R16	Fw	TAGCGCAGGAGGTCCACGACGTGCAAGGGTGTCGTGGTTGTGGGACCCGATC
	Rev	TAATACGACTCACTATAGGGCGGAAGGGTCCAGGCTAGGC
	Sec oligo	/5Atto565N/ACACCCTTGCACGTCGTGGACCTCCTGCGCTA/3Atto565N/
3R7	Fw	CACCGACGTCGCATAGAACGGAAGAGCGTGTGCCACAAGGGACACGCGTTCC
	Rev	TAATACGACTCACTATAGGGCGGAAGGGTCCAGGCTAGGC
	Sec oligo	/5Alex488N/CACACGCTCTTCCGTTCTATGCGACGTCGGTGagatgttt/3AlexF488N/
3R19	Fw	CACACGCTCTCCGTCTTGGCCGTGGTCGATCAGGTCCGTGCTGCGCTTTCTC
	Rev	TAATACGACTCACTATAGGGCGGAAGGGTCCAGGCTAGGC
	Sec oligo	/5Alex647N/TGATCGACCACGGCCAAGACGGAGAGCGTGTG/3AlexF647N/
69C	Fw	CACCGACGTCGCATAGAACGGAAGAGCGTGTGCGCTCGGTCTCCGTTCGTCTC
	Rev	TAATACGACTCACTATAGGGGGGCTAGGTACAGGGTTCAGC
	Sec oligo	/5Alex488N/CACACGCTCTTCCGTTCTATGCGACGTCGGTGagatgttt/3AlexF488N/
28B	Fw	TAGCGCAGGAGGTCCACGACGTGCAAGGGTGTCGCTCGGTCTCCGTTCGTCTC
	Rev	TAATACGACTCACTATAGGGGGGCTAGGTACAGGGTTCAGC
	Sec oligo	/5Atto565N/ACACCCTTGCACGTCGTGGACCTCCTGCGCTA/3Atto565N/

Supplementary Table 5. Primers and secondary oligos for Oligopaint probe sets.

For each Oligopaint probe set primer and secondary (sec) oligo sequences are given. In forward primer a site for secondary oligo annealing is underlined, while in reverse primer a T7 promoter sequence is underlined. Sequences for secondary oligos[19], adapted with a 5' and 3' fluorophore of interest, are provided using the modification codes from Integrated DNA Technologies (IDT).

SUPPLEMENTARY REFERENCES

1. Mackay, T.F. *et al*. The Drosophila melanogaster Genetic Reference Panel. *Nature* **482**, 173-8 (2012).
2. Danecek, P. *et al*. The variant call format and VCFtools. *Bioinformatics* **27**, 2156-8 (2011).
3. Crane, E. *et al*. Condensin-driven remodelling of X chromosome topology during dosage compensation. *Nature* **523**, 240-4 (2015).

4. Nora, E.P. *et al*. Targeted Degradation of CTCF Decouples Local Insulation of Chromosome Domains from Genomic Compartmentalization. *Cell* **169**, 930-944 e22 (2017).
5. Kerpedjiev, P. *et al*. HiGlass: web-based visual exploration and analysis of genome interaction maps. *Genome Biol*. **19**, 125 (2018).
6. Imakaev, M. *et al*. Iterative correction of Hi-C data reveals hallmarks of chromosome organization. *Nat. Methods* **9**, 999-1003 (2012).
7. Star, B. *et al*. Palindromic sequence artifacts generated during next generation sequencing library preparation from historic and ancient DNA. *PLoS One* **9**, e89676 (2014).
8. Golloshi, R., Sanders, J.T. & McCord, R.P. Iteratively improving Hi-C experiments one step at a time. *Methods* **142**, 47-58 (2018).
9. Zeitlinger, J. *et al*. Whole-genome ChIP-chip analysis of Dorsal, Twist, and Snail suggests integration of diverse patterning processes in the Drosophila embryo. *Genes Dev*. **21**, 385-90 (2007).
10. MacArthur, S. *et al*. Developmental roles of 21 Drosophila transcription factors are determined by quantitative differences in binding to an overlapping set of thousands of genomic regions. *Genome Biol*. **10**, R80 (2009).
11. Graveley, B.R. *et al*. The developmental transcriptome of Drosophila melanogaster. *Nature* **471**, 473-9 (2011).
12. Hug, C.B., Grimaldi, A.G., Kruse, K. & Vaquerizas, J.M. Chromatin Architecture Emerges during Zygotic Genome Activation Independent of Transcription. *Cell* **169**, 216-228 e19 (2017).
13. Sun, Y. *et al*. Zelda overcomes the high intrinsic nucleosome barrier at enhancers during Drosophila zygotic genome activation. *Genome Res*. **25**, 1703-14 (2015).
14. Xu, Z. *et al*. Impacts of the ubiquitous factor Zelda on Bicoid-dependent DNA binding and transcription in Drosophila. *Genes Dev* **28**, 608-21 (2014).
15. Negre, N. *et al*. A cis-regulatory map of the Drosophila genome. *Nature* **471**, 527-31 (2011).
16. Saunders, A., Core, L.J., Sutcliffe, C., Lis, J.T. & Ashe, H.L. Extensive polymerase pausing during Drosophila axis patterning enables high-level and pliable transcription. *Genes Dev*. **27**, 1146-58 (2013).
17. Ke, Y. *et al*. 3D Chromatin Structures of Mature Gametes and Structural Reprogramming during Mammalian Embryogenesis. *Cell* **170**, 367-381 e20 (2017).
18. Du, Z. *et al*. Allelic reprogramming of 3D chromatin architecture during early mammalian development. *Nature* **547**, 232-235 (2017).
19. Beliveau, B.J. *et al*. Single-molecule super-resolution imaging of chromosomes and in situ haplotype visualization using Oligopaint FISH probes. *Nat. Commun*. **6**, 7147 (2015).
