# Supplementary material for: The genome-wide multi-layered architecture of chromosome pairing in early Drosophila embryos
Source: Nat Commun. 2019 Oct 3;10:4486. doi: 10.1038/s41467-019-12211-8 (PMC6776651; doi:10.1038/s41467-019-12211-8)
Supplement: Supplementary file 3 — Reporting Summary [file 41467_2019_12211_MOESM3_ESM.pdf]

nature research

Corresponding author(s): Leonid A. Mirny, Chao-ting Wu

Last updated by author(s): Aug 23, 2019

Reporting Summary

Nature Research wishes to improve the reproducibility of the work that we publish. This form provides structure for consistency and transparency in reporting. For further information on Nature Research policies, see Authors & Referees and the Editorial Policy Checklist.

Statistics

For all statistical analyses, confirm that the following items are present in the figure legend, table legend, main text, or Methods section.

n/a	Confirmed	
☐	☒	The exact sample size (*n*) for each experimental group/condition, given as a discrete number and unit of measurement
☐	☒	A statement on whether measurements were taken from distinct samples or whether the same sample was measured repeatedly
☐	☒	The statistical test(s) used AND whether they are one- or two-sided<br>*Only common tests should be described solely by name; describe more complex techniques in the Methods section.*
☐	☒	A description of all covariates tested
☐	☒	A description of any assumptions or corrections, such as tests of normality and adjustment for multiple comparisons
☐	☒	A full description of the statistical parameters including central tendency (e.g. means) or other basic estimates (e.g. regression coefficient) AND variation (e.g. standard deviation) or associated estimates of uncertainty (e.g. confidence intervals)
☐	☒	For null hypothesis testing, the test statistic (e.g. F, t, r) with confidence intervals, effect sizes, degrees of freedom and P value noted<br>*Give P values as exact values whenever suitable.*
☒	☐	For Bayesian analysis, information on the choice of priors and Markov chain Monte Carlo settings
☐	☒	For hierarchical and complex designs, identification of the appropriate level for tests and full reporting of outcomes
☐	☒	Estimates of effect sizes (e.g. Cohen's *d*, Pearson's *r*), indicating how they were calculated

Our web collection on statistics for biologists contains articles on many of the points above.

Software and code

Policy information about availability of computer code

Data collection	Imaging data was acquired using the Zeiss LSM780 laser scanning confocal microscope with a 63x oil NA 1.40 lens at 1024x1024 resolution (Zeiss ZEN Software).
Data analysis	Data analysis was performed using: seqtk trimfq v.1.2-r94 (https://github.com/lh3/seqtk), bwa mem v.0.7.15, pairtools parse command line tool (https://github.com/mirnylab/pairtools), the cooler package (https://github.com/mirnylab/cooler), the package cooltools insulation (https://github.com/mirnylab/cooltools), peakdet: Peak detection using MATLAB, http://billauer.co.il/peakdet.html), ENCODE ChIP-seq pipeline (https://github.com/ENCODE-DCC/chip-seq-pipeline), ENCODE RNA-seq pipeline (https://github.com/ENCODE-DCC/long-rna-seq-pipeline/tree/master/dnanexus), Jupyter Notebooks, matplotlib, numpy, pandas, pyBedTools. We automated data analyses in command line interface using GNU Parallel. Details included in the Methods and Supplementary Note 1. The software used in this study is available at https://github.com/mirnylab/.

For manuscripts utilizing custom algorithms or software that are central to the research but not yet described in published literature, software must be made available to editors/reviewers. We strongly encourage code deposition in a community repository (e.g. GitHub). See the Nature Research guidelines for submitting code & software for further information.

Data

Policy information about availability of data

All manuscripts must include a data availability statement. This statement should provide the following information, where applicable:

- Accession codes, unique identifiers, or web links for publicly available datasets
- A list of figures that have associated raw data
- A description of any restrictions on data availability

The datasets generated and analyzed during this study are available in the Gene Expression Omnibus (GEO) repository under accession number GSE121255.

Field-specific reporting

Please select the one below that is the best fit for your research. If you are not sure, read the appropriate sections before making your selection.

☒ Life sciences ☐ Behavioural & social sciences ☐ Ecological, evolutionary & environmental sciences

For a reference copy of the document with all sections, see nature.com/documents/nr-reporting-summary-flat.pdf

Life sciences study design

All studies must disclose on these points even when the disclosure is negative.

Sample size	Sample size was not pre-determined. We used sample size commonly accepted for the type of experiments. For Hi-C experiments, we generated 2 biological replicates, and for FISH experiments, we counted at least 3 replicates with more than 100 nuclei/replicate.
Data exclusions	During Hi-C analyses, bins with low-coverage were excluded.
Replication	All attempts at replication were successful.
Randomization	Animals were not randomly allocated into experimental groups. Samples were treated in parallel with internal controls to minimize potential confounding effects.
Blinding	The investigators were not blinded during data collection and analysis.

Reporting for specific materials, systems and methods

We require information from authors about some types of materials, experimental systems and methods used in many studies. Here, indicate whether each material, system or method listed is relevant to your study. If you are not sure if a list item applies to your research, read the appropriate section before selecting a response.

Materials & experimental systems

n/a	Involved in the study	
☒	☐	Antibodies
☒	☐	Eukaryotic cell lines
☒	☐	Palaeontology
☐	☒	Animals and other organisms
☒	☐	Human research participants
☒	☐	Clinical data

Methods

n/a	Involved in the study	
☒	☐	ChIP-seq
☒	☐	Flow cytometry
☒	☐	MRI-based neuroimaging

Animals and other organisms

Policy information about studies involving animals; ARRIVE guidelines recommended for reporting animal research

Laboratory animals	F1 hybrid embryos (0-2hr, 2-4 hr, 6-8 hr, 10-12 hr) were obtained by mating DGRP-057 (virgin females) and 439 (males) strains (Bloomington stock numbers 29652 and 29658). To obtain Zld-depleted embryos, UAS-shRNA-zld (gift from the Rushlow laboratory) virgin females were crossed to the maternal triple driver Gal4 (MTD-Gal4, the Bloomington stock number 31777) males. Control samples were obtained by replacing MTD-Gal4 male flies with wild-type Canton S males.
Wild animals	This study did not involve wild animals.
Field-collected samples	This study did not involved samples collected from the field.
Ethics oversight	The Drosophila handling was in accordance with the Harvard Medical School guidelines.

Note that full information on the approval of the study protocol must also be provided in the manuscript.
