# Supplementary material for: The genome-wide multi-layered architecture of chromosome pairing in early Drosophila embryos
Source: Nat Commun. 2019 Oct 3;10:4486. doi: 10.1038/s41467-019-12211-8 (PMC6776651; doi:10.1038/s41467-019-12211-8)
Supplement: Supplementary file 4 — Description of Additional Supplementary Files [file 41467_2019_12211_MOESM4_ESM.pdf]

Description of Additional Supplementary File

File name: Supplementary Data 1

Description: Statistics summary for the haplotype-resolved mapping and duplicate removal using mammalian data.

The number of read pairs is provided for each replicate from Ke *et al*.[26] and Du *et al*.[25] datasets. Total raw read pairs ('Total') include unmapped read pairs with multiple or low score alignments comprising also reads without SNVs ('Total_unmapped'), and read pairs where only single side ('Total_ss_mapped') or both sides are mapped ('Total_mapped'). Total mapped read pairs contain non-duplicates and discarded PCR duplicates. Non-duplicates are subdivided into *cis* and *trans* read pairs.
